# Ternary Complexes of *cis*-(NH_3_)_2_PtCl_2_ (*cis*-DDP) With Guanosine (guo), Cytidine (cyd) and the Aminoacids Glycine (gly), L-Alanine (ala), L-2-Aminobutyric Acid (2-aba), L-Norvaline (nval) and L-Norleucine (nleu)

**DOI:** 10.1155/MBD.1997.339

**Published:** 1997

**Authors:** Erasmia Katsarou, Costas Charalambopoulos, Nick Hadjiliadis


					TERNARY COMPLEXES OF cis-(NH3)2PtCI2 (cis-DDP) WITH
GUANOSINE (guo), CYTIDINE (cyd) AND THE AMINOACIDS
GLYCINE (gly), L-ALANINE (ala), L-2-AMINOBUTYRIC ACID (2-aba),
L-NORVALINE (nval) AND L-NORLEUCINE (nleu)
Erasmia Katsarou, Costas Charalambopoulos and Nick Hadjiliadis*
Pages 57-63. The first six tables were omitted. They are given below.
339
Vol. 4, No. 6, 1997 Ternary Complexes ofcis-(NH3)2PtCl2(cis-DDP) with Guanosine(guo), Cytidine
0
ZIZ
o
00(0 O) D'.-
CzC
zzz
8ooo
ZZZZZZZZZ
z o
ZZ Z Z Z
340
Erasmia Katsarou et al. Metal Based Drugs
0 149 eO O0 0 LO t"O
I"-. CO O CO
t'- I'-.
341
Vol. 4, No. 6, 1997 Ternary Complexes ofcis-(NH3)2PtCl2(cis-DDP) with Guanosine(guo), Cytidine
EEEEE
zZZZZZZZZ
342
Erasmia Katsarou et al. Metal Based Drugs
c
C
0
C
0
I21
0 I'-. CO CO 0,1 0
CO 0 O
g g g g g
o o o4 a0
o o o o)
I-,. I'-. I' I'-.. o I',-.
z o
Z Z
0 0 0 0 0
o0
: o o
g g g g g g
E E E E E
343

Table I: $^1$H-NMR Chemical Shifts of the Compounds in $D_2O$ (ppm)

| Compounds | pD | $H_8$ | $H_6$ | $H_5$ | α-CH | β-CH | γ-CH | δ-CH | ε-CH |
|---|---|---|---|---|---|---|---|---|---|
| *cis*-[$(NH_3)_2$Pt(gly)]$NO_3$ | 5.5 | | | | 3.642(s) | | | | |
| *cis*-[$(NH_3)_2$Pt(ala)]$NO_3$ | 5.5 | | | | 3.778(q) | 1.475(t) | | | |
| *cis*-[$(NH_3)_2$Pt(2-aba)]$NO_3$ | 5.5 | | | | 3.649(t) | 1.902(m) | 1.093(t) | | |
| *cis*-[$(NH_3)_2$Pt(nval)]$NO_3$ | 5.5 | | | | 3.733(t) | 1.865(m) | 1.573(m) | 1.003(t) | |
| *cis*-[$(NH_3)_2$Pt(nleu]$NO_3$ | 5.5 | | | | 3.704(t) | 1.893(m) | 1.588(m) | 1.427(m) | 0.968(t) |
| *cis*-[$(NH_3)_2$Pt(guo)(gly)]$NO_3$ | 5.6 | 8.604(s) | | | 3.145(s) | | | | |
| *cis*-[$(NH_3)_2$Pt(guo)(ala)]$NO_3$ | 5.6 | 8.573(s) | | | 3.128(q) | 1.311(d) | | | |
| *cis*-[$(NH_3)_2$Pt(guo)(2-aba)]$NO_3$ | 5.6 | 8.574(s) | | | 2.999(s,br) | 1.663(m) | 0.818(t) | | |
| *cis*-[$(NH_3)_2$Pt(guo)(nval)]$NO_3$ | 5.6 | 8.589(s) | | | 3.036(t) | 1.560(m) | 1.156(m) | 0.796(t) | |
| *cis*-[$(NH_3)_2$Pt(guo)(nleu]$NO_3$ | 5.6 | 8.599(s) | | | 3.025(s,br) | 1.552(m) | 1.035(m) | 1.035(m) | 0.757(t) |
| guo | | 7.987(s) | | | | | | | |
| *cis*-[$(NH_3)_2$Pt(cyd)(gly)]$NO_3$ | 5.5 | | 7.966, 7.953. 7.937(t) | 6.154, 6.142(d) | 3.258(t) | | | | |
| *cis*-[$(NH_3)_2$Pt(cyd)(ala)]$NO_3$ | 5.5 | | 7.953, 7.944, 7.940, 7.931(dd) | 6.156, 6.142, 6.128 (t) | 3.378(q) 3.288(q) | 1.348(d) | | | |
| *cis*-[$(NH_3)_2$Pt(cyd)(2-aba)]$NO_3$ | 5.5 | | 7.957, 7.948. 7.945, 7.936(dd) | 6.166, 6.153, 6.145, 6.132(dd) | 3.314(t) 3.173(t) | 1.692(m) | 0.909(t) | | |
| *cis*-[$(NH_3)_2$Pt(cyd)(nval)]$NO_3$ | 5.5 | | 7.965. 7.955, 7.953, 7.943(dd) | 6.168. 6.155, 6.147, 6.135(dd) | 3.334(t) 3.199(t) | 1.637(m) | 1.237(m) | 0.876(t) | |
| *cis*-[$(NH_3)_2$Pt(cyd)(nleu)]$NO_3$ | 5.5 | | 7.974. 7.967, 7.955, 7.948(dd) | 6.177. 6.156, 6.142(t) | 3.327(t) 3.193(t) | 1.673(m) | 1.250(m) | 1.250(m) | 0.842(t) |
| cyd | 5.5 | | 7.864(d) | 6.073(d) | | | | | |

Table II: $^{13}$C-NMR Chemical Shifts of the Compounds in $D_2O$ (ppm)

| Compounds | $C_2$ | $C_4$ | $C_5$ | $C_6$ | $C_8$ | $C_{1'}$ | $C_{2'}$ | $C_{3'}$ | $C_{4'}$ | $C_{5'}$ | $C_\alpha$ | $C_\beta$ | $C_\gamma$ | $C_\delta$ | Cε | $C_o$ |
|---|---|---|---|---|---|---|---|---|---|---|---|---|---|---|---|---|
| gly | | | | | | | | | | | 44.32 | | | | | 175.28 |
| ala | | | | | | | | | | | 53.50 | 19.03 | | | | 178.61 |
| 2-aba | | | | | | | | | | | 58.73 | 26.52 | 11.37 | | | 177.65 |
| nval | | | | | | | | | | | 57.54 | 35.37 | 20.68 | 15.76 | | 177.90 |
| nleu | | | | | | | | | | | 57.62 | 32.87 | 29.18 | 24.46 | 15.77 | 177.89 |
| *cis* -[$(NH_3)_2$Pt(gly)]NO | | | | | | | | | | | 49.03 | | | | | 191.71 |
| *cis* -[$(NH_3)_2$Pt(ala)]$NO_3$ | | | | | | | | | | | 56.64 | 21.01 | | | | 192.00 |
| *cis* -[$(NH_3)_2$Pt(2-aba)]$NO_3$ | | | | | | | | | | | 61.73 | 28.53 | 11.66 | | | 192.11 |
| *cis* -[$(NH_3)_2$Pt(nval)]$NO_3$ | | | | | | | | | | | 60.33 | 37.31 | 20.74 | 15.70 | | 192.41 |
| *cis* -[$(NH_3)_2$Pt(nleu)]$NO_3$ | | | | | | | | | | | 60.51 | 34.90 | 29.39 | 24.48 | 15.88 | 187.67 |
| *cis* -[$(NH_3)_2$Pt(guo)(gly)]$NO_3$ | 157.40 | 153.50 | 117.44 | 159.72 | 142.83 | 91.64 | 72.78 | 76.78 | 88.35 | 63.80 | 51.22 | | | | | 178.22 |
| *cis* -[$(NH_3)_2$Pt(guo)(ala)]$NO_3$ | 157.42 | 153.51 | 117.36 | 159.72 | 142.82 | 91.63 | 72.72 | 76.78 | 88.31 | 63.74 | 59.15 | 22.42 | | | | 181.53 |
| *cis* -[$(NH_3)_2$Pt(guo)(2-aba)]$NO_3$ | 157.48 | 153.57 | 117.21 | 159.78 | 142.78 | 91.52 | 72.72 | 76.84 | 88.37 | 63.78 | 64.94 | 29.75 | 11.66 | | | 180.46 |
| *cis* -[$(NH_3)_2$Pt(guo)(nval)]$NO_3$ | 157.50 | 153.60 | 118.85 | 159.77 | 142.88 | 91.56 | 72.86 | 76.87 | 88.44 | 63.82 | 63.57 | 38.67 | 20.97 | 15.68 | | 180.73 |
| *cis* -[$(NH_3)_2$Pt(guo)(nleu)]$NO_3$ | 157.52 | 153.61 | 118.80 | 159.79 | 142.94 | 91.63 | 72.87 | 76.96 | 88.45 | 63.86 | 63.69 | 36.15 | 29.43 | 15.86 | 12.96 | 180.74 |
| guo[20] | 153.57 | 151.23 | 116.57 | 156.70 | 135.54 | 86.28 | 70.28 | 73.62 | 85.12 | 61.67 | | | | | | |
| *cis* -[$(NH_3)_2$Pt(cyd)(gly)]$NO_3$ | 157.04 | 168.23 | 98.05 | 145.32 | | 94.28 | 72.00 | 77.00 | 86.96 | 63.43 | 51.89 | | | | | 178.64 |
| | | | | 144.99 | | 93.94 | | 76.80 | | | | | | | | 178.22 |
| *cis* -[$(NH_3)_2$Pt(cyd)(ala)]$NO_3$ | 157.00 | 168.24 | 98.11 | 145.32 | | 94.33 | 72.04 | 76.96 | 87.03 | 63.46 | 59.79 | 22.68 | | | | 181 45 |
| | 156.88 | 168.13 | 98.04 | 145.06 | | 93.90 | 71.98 | 76.85 | 86.92 | | 59.53 | | | | | |
| *cis* -[$(NH_3)_2$Pt(cyd)(2-aba)]$NO_3$ | 157.01 | 168.22 | 98.16 | 145.38 | | 94.37 | 72.10 | 77.05 | 87.11 | 63.48 | 65.55 | 29.99 | 11.76 | | | 180.22 |
| | 156.86 | 168.09 | | 145.07 | | 93.71 | 71.99 | 76.23 | 86.96 | | 65.18 | 29.65 | 11.65 | | | |
| *cis* -[$(NH_3)_2$Pt(cyd)(nval)]$NO_3$ | 157.00 | 168.23 | 98.14 | 145.39 | | 94.38 | 72.09 | 77.05 | 87.09 | 63.47 | 64.25 | 38.91 | 21.06 | 15.91 | | 180.50 |
| | 156.87 | 168.09 | 98.02 | 145.07 | | 93.80 | 71.99 | 76.83 | 86.96 | | 63.95 | 38.85 | | 15.86 | | |
| *cis* -[$(NH_3)_2$Pt(cyd)(nleu)]$NO_3$ | 157.01 | 168.22 | 98.13 | 145.40 | | 94.36 | 72.07 | 77.08 | 87.08 | 63.44 | 64.43 | 36.44 | 29.85 | 24.65 | 15.95 | 180.51 |
| | 156.80 | 168.08 | 98.02 | 145.08 | | 93.82 | 71.98 | 76.85 | 86.96 | | 64.10 | 36.36 | 29.78 | 24.59 | | |
| cyd | 159.85 | 168.57 | 98.92 | 144.60 | | 93.17 | 72.12 | 76.79 | 86.63 | 63.57 | | | | | | |

Table III: Characteristic IR bands of the complexes ($cm^{-1}$)

| Compounds | $\nu$(C=O)nucl+ $\nu^{as}$($COO^-$)amac | $\delta$NH($NH_2$,$NH_3$) | $\nu$(C=C,C=N) | ring breathing | sugar conformation |
|---|---|---|---|---|---|
| *cis*-[$(NH_3)_2$Pt(guo)(gly)]$NO_3$ | 1695(s,br) | 1625(s,br) | 1585(s), 1495(m) | 690(m) | 822(m), 795(w) |
| *cis*-[$(NH_3)_2$Pt(guo)(ala)]$NO_3$ | 1714(s) | 1634(s) | 1585(s), 1505(m) | 699(m) | 825(m), 802(w) |
| *cis*-[$(NH_3)_2$Pt(guo)(2-aba)]$NO_3$ | 1696(s,br) | 1634(s) | 1588(s), 1498(m) | 692(m) | 825(m), 804(w) |
| *cis*-[$(NH_3)_2$Pt(guo)(nval)]$NO_3$ | 1686(s,br) | 1633(s) | 1591(s), 1502(m) | 701(m) | 825(m), 802(w) |
| *cis*-[$(NH_3)_2$Pt(guo)(nleu)]$NO_3$ | 1691(s) | 1636(s) | 1588(s), 1501(m) | 701(m) | 825(m), 802(w) |
| *cis*-[$(NH_3)_2$Pt(cyd)(gly)]$NO_3$ | 1651(s) | 1635(s), 1538(s) | 1622(m), 1505(m) | 791(w), 777(m) | |
| *cis*-[$(NH_3)_2$Pt(cyd)(ala)]$NO_3$ | 1652(m) | 1645(m),1540(sh) | 1621(s), 1505(m) | 791(w), 772(m) | |
| *cis*-[$(NH_3)_2$Pt(cyd)(2-aba)]$NO_3$ | 1656(s) | 1649(s), 1537(m) | 1618(m), 1504(m) | 793(m), 776(m) | |
| *cis*-[$(NH_3)_2$Pt(cyd)(nval)]$NO_3$ | 1657(s) | 1645(s), 1538(m) | 1615(m), 1505(m) | 791(m), 774(m) | |
| *cis*-[$(NH_3)_2$Pt(cyd)(nleu)]$NO_3$ | 1657(s) | 1646(s), 1537(m) | 1617(m), 1505(m) | 791(m), 775(m) | |

Table IV: Difference in chemical shifts $\Delta\delta$ (ppm) of the protons of the zwitterionic forms of the aminoacids and the ternary complexes coordinated ones

| $\Delta\delta$ | $H_\alpha$ | $H_\beta$ | $H_\gamma$ | $H_\delta$ | $H_\varepsilon$ |
|---|---|---|---|---|---|
| $\delta_{2\text{-}aba}-\delta_{tern\ complex(guo)}$ | 0.711 | 0.236 | 0.210 | | |
| $\delta_{nval}-\delta_{tern\ complex(guo)}$ | 0.698 | 0.273 | 0.239 | 0.153 | |
| $\delta_{nleu}-\delta_{tern.complex(guo)}$ | 0.702 | 0.297 | 0.270 | 0.220 | 0.143 |
| $\delta_{2\text{-}aba}-\delta_{tern\ complex(cyd)}$ | 0.537 0.396 | 0.207 | 0.139 | | |
| $\delta_{nval}-\delta_{tern.complex(cyd)}$ | 0.535 0.400 | 0.196 | 0.158 | 0.093 | |
| $\delta_{nleu}-\delta_{tern.complex(cyd)}$ | 0.534 0.400 | 0.176 | 0.165 | 0.105 | 0.058 |

Table V: Difference in chemical shifts (ppm) of the terminal methyl groups of the anionc forms of the aminoacids and the ternary complexes coordinated ones

| $\Delta\delta$ | ala | 2-aba | nval | nleu |
|---|---|---|---|---|
| $\delta_{amac}-\delta_{tern.complex(guo)}$ | -0.09 | 0.094 | 0.107 | 0.130 |
| $\delta_{amac}-\delta_{tern.complex(cyd)}$ | -0.128 | 0.003 | 0.027 | 0.045 |

Table VI: $^{1}$H-NMR Chemical Shifts, Coupling Constants and percentages of gg, $^{3}$E and anti sugar conformations

| Compounds | pD | $H_{1'}$ | $H_{2'}$ | $H_{3'}$ | $H_{4'}$ | $H_{5'5''}$ | $J_{1'2'}$ | $J_{2'3'}$ | $J_{3'4'}$ | $J_{4'5'}$ | $J_{4'5''}$ | *gg* % | $^{3}E$ % | Keq ($^{3}E/^{2}E$) | *anti* % |
|---|---|---|---|---|---|---|---|---|---|---|---|---|---|---|---|
| *cis*-[$(NH_3)_2$Pt(guo)(gly)]$NO_3$ | 5.6 | 5.985, 5 973(d) | 4.710(t) | 4.417(t) | 4.253(q) | 3.888(m) | 4.80 | 4.86 | 3.98 | 2.94 | 4.12 | 68 | 45 | 0.82 | 51 |
| *cis*-[$(NH_3)_2$Pt(guo)(ala)]$NO_3$ | 5.6 | 5.987 5.979(d) | 4.700(t) | 4.416(t) | 4.259(q) | 3.899(m) | 5.07 | 4.88 | 4.09 | 2.88 | 4.07 | 69 | 45 | 0.82 | 53 |
| *cis*-[$(NH_3)_2$Pt(guo)(2-aba)]$NO_3$ | 5.6 | 5.968 5.959(d) | 4.706(t) | 4.417(t) | 4.263(q) | 3.893(m) | 5.13 | 4.81 | 3.92 | 2.84 | 4.06 | 70 | 43 | 0.75 | 52 |
| *cis*-[$(NH_3)_2$Pt(guo)(nval)]$NO_3$ | 5.6 | 5.998 5.985(d) | 4.702(t) | 4.416(t) | 4.265(q) | 3.895(m) | 5.13 | 4.76 | 4.04 | 2.90 | 4.11 | 69 | 44 | 0.78 | 53 |
| *cis*-[$(NH_3)_2$Pt(guo)(nleu]$NO_3$ | 5.6 | 5.993 5.984(d) | 4.698(t) | 4.410(t) | 4.261(q) | 3.890(m) | 5.12 | 4.82 | 4.08 | 2.94 | 4.11 | 68 | 44 | 0.78 | 53 |
| guo[a] | | 5.895(d) | 4.712(t) | 4.394(t) | 4.215(m) | 3.845(m) | 5.90 | 5.20 | 3.70 | 3.20 | 3.80 | 69 | 38 | 0.61 | 51 |
| *cis*-[$(NH_3)_2$Pt(cyd)(gly)]$NO_3$ | 5.5 | 5.931 5.925 5.903 5.896(dd) | 4.341(q) | 4.209(t) | 4.161(m) | 3.890(m) | 3.61 | | 5.05 | 2.72 | 4.74 | 64 | 58 | 1.38 | |
| *cis*-[$(NH_3)_2$Pt(cyd)(ala)]$NO_3$ | 5.5 | 5.939 5.933 5.897 5.891(dd) | 4.352(q) | 4.221(q) | 4.164(m) | 3.888(m) | 3.68 | | 5.37 | 2.73 | 4.27 | 69 | 59 | 1.44 | |
| *cis*-[$(NH_3)_2$Pt(cyd)(2-aba)]$NO_3$ | 5.5 | 5.948 5.942 5.896 5.890(dd) | 4.350(m) | 4.218(q) | 4.165(m) | 3.886(m) | 3.69 | | | 2.75 | 4.32 | 68 | | | |
| *cis*-[$(NH_3)_2$Pt(cyd)(nval)]$NO_3$ | 5.5 | 5.944 5.938 5.891 5.885(dd) | 4.344(m) | 4.215(q) | 4.162(m) | 3.887(m) | 3.66 | | 5.89 | 2.69 | 4.32 | 69 | 61 | 1.56 | |
| *cis*-[$(NH_3)_2$Pt(cyd)(nleu)]$NO_3$ | 5.5 | 5.947 5.927 5.893 5.884(dd) | 4.344(m) | 4.219(q) | 4.168(m) | 3.887(m) | 3.72 | | | 3.04 | 4.36 | 65 | | | |
| cyd | 5.5 | 5.920(d) | 4.331(t) | 4.229(t) | 4.156(q) | 3.898(m) | | | | 2.24 | 4.32 | 73 | 60[b] | 1.50 | |

[a]Taken from ref.35, [b]Taken from ref.41.
